# Scan-Rate-Dependent Ion Current Rectification in Bipolar Interfacial Nanopores

**DOI:** 10.3390/mi15091176

**Published:** 2024-09-23

**Authors:** Xiaoling Zhang, Yunjiao Wang, Jiahui Zheng, Chen Yang, Deqiang Wang

**Affiliations:** 1 School of Smart Health, Chongqing Polytechnic University of Electronic Technology, Chongqing 401331, China; 2 Chongqing Institute of Green and Intelligent Technology, Chinese Academy of Sciences, Chongqing 400714, China; wangyunjiao@cigit.ac.cn; 3 Key Laboratory of Biorheological Science and Technology, Ministry of Education and Bioengineering College, Chongqing University, Chongqing 400044, China; jiahuizheng@cqu.edu.cn (J.Z.); yangchencq@cqu.edu.cn (C.Y.)

**Keywords:** ion current rectification, interfacial nanopore, bipolar, memory effects

## Abstract

This study presents a theoretical investigation into the voltammetric behavior of bipolar interfacial nanopores due to the effect of potential scan rate (1–1000 V/s). Finite element method (FEM) is utilized to explore the current–voltage (I–V) properties of bipolar interfacial nanopores at different bulk salt concentrations. The results demonstrate a strong impact of the scan rate on the I–V response of bipolar interfacial nanopores, particularly at relatively low concentrations. Hysteresis loops are observed in bipolar interfacial nanopores under specific scan rates and potential ranges and divided by a cross-point potential that remains unaffected by the scan rate employed. This indicates that the current in bipolar interfacial nanopores is not just reliant on the bias potential that is imposed but also on the previous conditions within the nanopore, exhibiting history-dependent or memory effects. This scan-rate-dependent current–voltage response is found to be significantly influenced by the length of the nanopore (membrane thickness). Thicker membranes exhibit a more pronounced scan-rate-dependent phenomenon, as the mass transfer of ionic species is slower relative to the potential scan rate. Additionally, unlike conventional bipolar nanopores, the ion current passing through bipolar interfacial nanopores is minimally affected by the membrane thickness, making it easier to detect.

## 1. Introduction

Nanofluidic memristors, known as memory resistors constructed within nanoconfined fluidic systems, demonstrate ion conductivity that is dependent on the system’s history [1,2,3]. This implies that the response signal of nanofluidic memristors is not just impacted by outside stimuli but also by the system’s past or prior states. Over the past few years, they have gained significant attention as essential electrical components for brain-inspired neuromorphic computing [4,5,6,7,8,9]. Nanofluidic memristors have been used to emulate the functions of neurons and synapses [3,10,11] and have shown great potential in constructing implantable biohybrid devices [12].

Three distinct types of nanofluidic memristors, characterized by their voltage-responsive dynamics, have been identified based on different system mechanisms [12]. These include memristors based on wall–fluid interaction [1,13], interfacial movement [14,15], and structural deformation [16,17]. The first type relies on the hysteresis nature of nonlinear ion effects within confined nanochannels or nanoslits, such as ion current rectification (ICR). For example, nanofluidic memristors stemmed from the impedance study of glass nanopipettes [18]. Using Poisson–Nernst–Planck equations, Momotenko et al. [19] reported the diode-like behavior of conical nanopores at different potential scan rates and the ICR phenomenon is scan-rate-dependent. Wang et al. [20] reported the memristive and memcapacitive ion transport in single conical nanopores. Guerrette et al. [21] showed that the I–V response of quartz nanopipettes is dependent on the bias scan rate. In addition to nanopipettes/conical nanopores, the memristive effect is also found in other confined spaces. Conroy et al. [22] investigated hysteresis effects and the relationship between scan rate and current response in a bipolar membrane. The reason behind this dependency is that, due to the refined mobility of ions, the movement of ionic species within the nanopore may be slower compared to rapid changes in voltage when they redistribute within charged nanopores under applied potentials. These findings suggest that these nanofluidic devices could potentially be used in memristors because the ion rearrangement within these confined spaces is time-dependent. Additionally, owing to other nonlinear ion effects, such as ultrafast ion transport and ion selectivity generated by the enhanced confinement effect in 2D channels, 2D nanofluidic memristors have also been proposed [13,23,24]. However, it should be noted that the design of nanofluidic memristors is still in its early stages [12].

Among these nonlinear ion effects, the ICR phenomenon has been observed in different systems, such as nanopores with heterogeneous interfaces [25,26,27,28], bullet-shaped nanopores [29,30], and conical nanopores/nanopipettes [31,32,33,34,35]. The unequal distribution of surface charge density on nanopore/nanochannel walls and the asymmetric confined structure have typically been considered as the factors responsible for current rectification, resulting in a nonlinear I–V response. Besides the nanofluidic memristors, the rectified nanofluidic systems have been experimentally proven to amplify current, conductance, and osmotic energy conversion performance [36,37,38,39,40,41,42].

Based on the causes of the ICR phenomenon, we propose a new method for generating nonlinear I–V using interfacial nanopores. Interfacial nanopores are strictly two-dimensional nanopores defined by parallelepipeds at their intersection, without any discernible thickness. They offer the shortest possible channel length, exceptional mechanical stability, and small noise. The fabrication of interfacial nanopores does not require extremely high precision and can be easily processed into an array. According to Luan et al. [43], membrane thickness has little effect on the ionic current passing through interfacial nanopores. To induce the nonlinear I–V or ICR phenomenon, two common materials can be used to process and form interfacial nanopores, creating a difference in surface charges between the upper and lower nanochannels. This nonlinear ion effect has potential applications in the fabrication of nanofluidic memristors [12]. Therefore, in this study, we investigate a detailed understanding of the ICR mechanism in bipolar interfacial nanopores at various potential scan rates by FEM simulations. We utilize the transient form of the Poisson–Nernst–Planck (PNP) equations and Navier–Stokes (NS) equations to study the rectification behavior of interfacial nanopores. In a bipolar interfacial nanopore, when the membrane thickness (2 × height of the nanochannel) is sufficiently long, the redistribution speed of ions cannot keep up with the speed of voltage change, leading to the occurrence of the memristive effect. Memory effects are observed in both bipolar interfacial nanopores and conventional bipolar nanopores, indicating that this phenomenon is not influenced by the shape of the nanopore. Furthermore, compared to conventional bipolar nanopores, the membrane thickness of the interfacial nanopore has a small impact on the current. As a result, increasing the membrane thickness can still yield a larger current. These properties of bipolar interfacial nanopores offer the possibility to modify the memory effect of this system by simply adjusting the surface charge density of the upper and lower nanochannels. Thus, it provides a potential new approach for the design of nanofluidic memristors.

## 2. Theoretical Model

**Figure 1:**
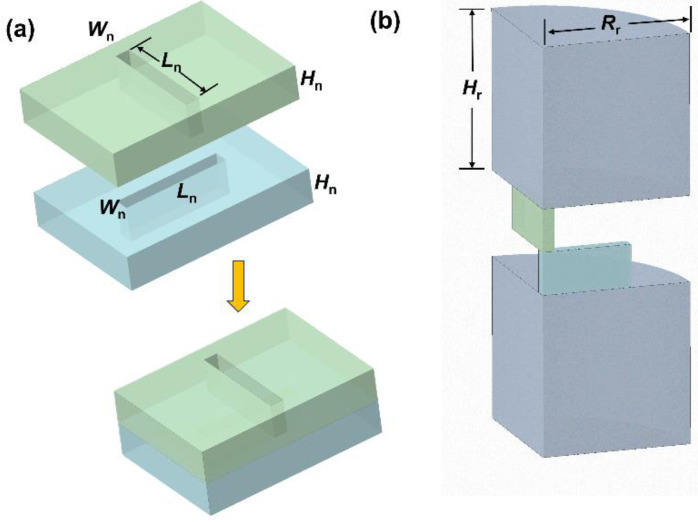
Illustration of the bipolar interfacial nanopore: (**a**) The structure of the interfacial nanopore. Two nanochannels with length *L*_n_, width *W*_n_, and height *H*_n_ form an interfacial nanopore. (**b**) Simulation domain. The gray parts represent the two reservoirs, the blue part represents the lower half of the bipolar interfacial nanopore, and the green part represents the upper half of the bipolar interfacial nanopore.

Two identical crossing cuboids with length *L*_n_, width *W*_n_, and height *H*_n_ constitute the interfacial nanopore, as illustrated schematically in Figure 1a, and on both sides are two reservoirs with a length *L*_r_ and a radius *R*_r_, forming the simulation domain (Figure 1b). It is selected to use the three-dimensional Cartesian coordinate system (*x*, *y*, *z*). *x*, *y*, and *z* are, respectively, pointed in the direction of the length, width, and height of the lower cuboid, and the origin is situated in the center of the interface between the two crossing cuboids. The whole domain is stuffed with KCl electrolyte solution. Two ionic species, K^+^ and Cl^−^, are considered in the simulations. The model based on PNP and NS equations in transient form is applied to study the time-dependent electrostatics, hydrodynamics, and transport of ionic mass [44,45,46]:

(1) Poisson–Nernst–Planck equation

(1) $$-\varepsilon_{0}\varepsilon_{f}\nabla^{2}\phi =F\left(c_{1}z_{1}+c_{2}z_{2}\right)$$

(2) $$\frac{\partial c_{i}}{\partial t}+\nabla \cdot J_{i}=0$$

The flux:

(3) $$J_{i}=uc_{i}-D_{i}\nabla c_{i}-z_{i}\frac{D_{i}}{RT}Fc_{i}\nabla \phi \;\ldots \;(i=1,2)$$

Here, *ε*_0_ represents the permittivity of vacuum; *ε_f_* denotes the relative permittivity of the KCl electrolyte solution; *z_i_*, *c_i_*, *D_i_*, and *ϕ* correspond to the valence, the concentration, the diffusion coefficient of ionic species (*i* = 1 for K^+^ and 2 for Cl^−^), and the electric potential, respectively. *R* signifies the universal gas constant, *T* represents the absolute temperature, and *F* denotes the Faraday constant.

(2) Navier–Stokes equation

(4) $$\rho \frac{\partial u}{\partial t}=-\nabla p+\mu \nabla^{2}u+F$$

(5) ∇·u=0

(6) $$F=-F\left(c_{1}z_{1}+c_{2}z_{2}\right)\nabla \phi$$

where *p*, *μ*, and **u** represent the hydrodynamic pressure, fluid viscosity, and fluid velocity vector, respectively. The interaction between the electric field and net charge density leads to the generation of the electrostatic force **F** within the nanopore, resulting in electroosmotic flow (EOF). The contribution of inertial terms can be disregarded due to the negligible Reynolds numbers associated with the EOFs passing through nanopores.

As summarized in Table S1, the aforementioned equations are solved using the boundary conditions as follows [19,25,45]:

At the reservoirs’ ends, the ionic concentrations are kept at the values of the bulk salt concentrations (*c_i_* = *C_i_*_0_), and the electric potentials are *ϕ* (bottom end) = Δ*ϕ* and *ϕ* (top end) = 0. The bias potential that is imposed is modulated as Δ*ϕ* = −Δ*ϕ*_0_ + *νt* (forward scan) and Δ*ϕ* = Δ*ϕ*_0_ − *νt* (backward scan), where Δ*ϕ*_0_ is the initial bias potential; thus, the bias potential that is imposed is scanned from negative to positive values and then from positive to negative values. Each reservoir end is governed by an outlet boundary condition with no external pressure gradient. The side walls of the reservoirs are subjected to the boundary conditions of zero normal ionic fluxes ($n\cdot N_{i}=0)$, zero surface charge (n·∇ϕ=0), and slip for the flow field. The walls of the nanopore (membrane) are ion-impenetrable (zero normal ionic fluxes), and a nonslip boundary condition is applied on the entire nanopore surface. The upper and lower halves of the interfacial nanopore have fixed surface charge densities of *σ_w1_* ($-n\cdot \nabla \phi =\sigma_{w1}/\left(\varepsilon_{0}\varepsilon_{f}\right)$) and *σ_w2_* ($-n\cdot \nabla \phi =\sigma_{w2}/\left(\varepsilon_{0}\varepsilon_{f}\right)$), respectively, where **n** denotes the unit normal vector. Note that in practical nanofluidic systems, the nanopore/nanochannel wall surface exhibits charge regulation properties [41,47,48,49], which could be further investigated in the future. For computational efficiency, it is assumed that surface charges are fixed and present on the membrane only near the nanochannels (Figure S1).

The induced ionic current flowing through the bipolar interfacial nanopore can be obtained by [50,51,52]:

(7) $$I=4\int F\left(z_{1}J_{1}+z_{2}J_{2}\right)\cdot ndS$$

*S* denotes the surface of either end of the reservoirs (either the anode or cathode). To reduce the amount of computations, we only calculate a quarter of the structure of the system according to the symmetry of the simulation domain (Figure 1b), that is, the actual ionic current values are four times the integration obtained ionic current.

## 3. Results and Discussion

A commercial finite element program (COMSOL Multiphysics, version 5.4) is used to solve the model numerically. The PNP + NS model has also been used to solve similar problems related to ion transport through nanopores [44,53,54]. A fine mesh is applied to the surface of the nanochannels to make certain that the obtained results are fully convergent and mesh-independent. The following parameters are adopted: μ = 1 × 10^−3^ Pa·s, ρ = 1 × 10^3^ kg/m^3^, *ε*_0_ = 8.854 × 10^−12^ F/m, *ε*_f_ = 80, *R* = 8.31 J/(K mol), *T* = 300 K, *F* = 96,490 C/mol, D_1_(K^+^) = 1.96 × 10^−9^ m^2^/s, D_2_(Cl^−^) = 2.03 ×10^−9^ m^2^/s [45], and Δ*ϕ*_0_ = 0.6 V. The applied potential bias increases from −0.5 V to 0.5 V and then decreases from 0.5 V to −0.4 V. Note that we remove the values at t = 0 because there is a sudden change in these values. The sizes of the reservoirs are *R_r_* = 200 nm and *H_r_* = 200 nm. The length of the nanochannels is *L*_n_ = 500 nm, while the width *W*_n_ and height *H*_n_ can be adjusted as needed. The thickness of the whole membrane is 2 × *H*_n_. The lower half of the bipolar interfacial nanopore carries negative surface charges with a density of −1 mC/m^2^, and the upper half carries positive surface charges with a density of 1 mC/m^2^. In the following discussions, influences of the scan rate, the bulk salt concentration, *C*_KCl_, and dimensions of the nanochannels on the I–V response are investigated comprehensively for bipolar interfacial nanopores.

### 3.1. Effect of the Potential Scan Rate

**Figure 2:**
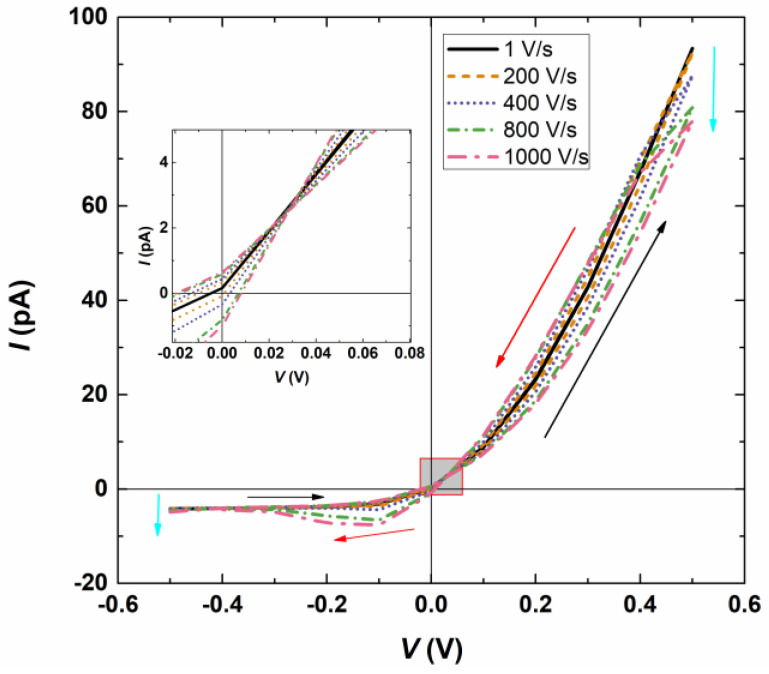
I–V responses as the scan rates change from 1 V/s to 1000 V/s when *C*_KCl_ = 0.5 mM. The forward (backward) direction of the potential scan is shown by the black (red) arrows. The current is indicated by the cyan arrows with an increasing scan rate.

Figure 2 illustrates the I–V responses across the bipolar interfacial nanopore (*W*_n_ = 40 nm and *H*_n_ = 800 nm) as the scan rates change from 1 V/s to 1000 V/s while maintaining a bulk salt concentration of 0.5 mM. The rectified I–V responses are a result of the surface-charge asymmetry within the bipolar interfacial nanopore. In the case of a bipolar interfacial nanopore, with half of the surface negatively charged and the other half positively charged, applying a positive bias potential on the side of the negatively charged membrane creates a state with high conductivity, while a state with low conductivity is established otherwise. It can be seen that the current amplitudes display variation at various scan rates. As the scan rate increases, the amplitude of the current significantly decreases in the high conductivity state and slightly increases in the low conductivity state, similar to phenomena observed in single conical nanopores [20]. This change in resistance over time aligns with the memristive effects discussed in electronics [20]. Moreover, the ionic current amplitudes exhibit differences and intersect when scanned in opposite directions (from −0.5 to +0.5 V versus from +0.5 to −0.4 V). Specifically, under specific scan rates and potential ranges, hysteresis loops are observed in bipolar interfacial nanopores. At the low and high conductivity states, the two hysteresis loops are divided by a cross-point potential that remains unaffected by the scan rate employed and is approximately 30 mV, as depicted in the magnified image in Figure 2. Wang et al. [20] also reported a similar cross-point potential in single conical nanopores. Additionally, it is evident that the loop area expands with increasing scan rates. These characteristics may provide a foundation for the utilization of bipolar interfacial nanopores in memristors.

**Figure 3:**
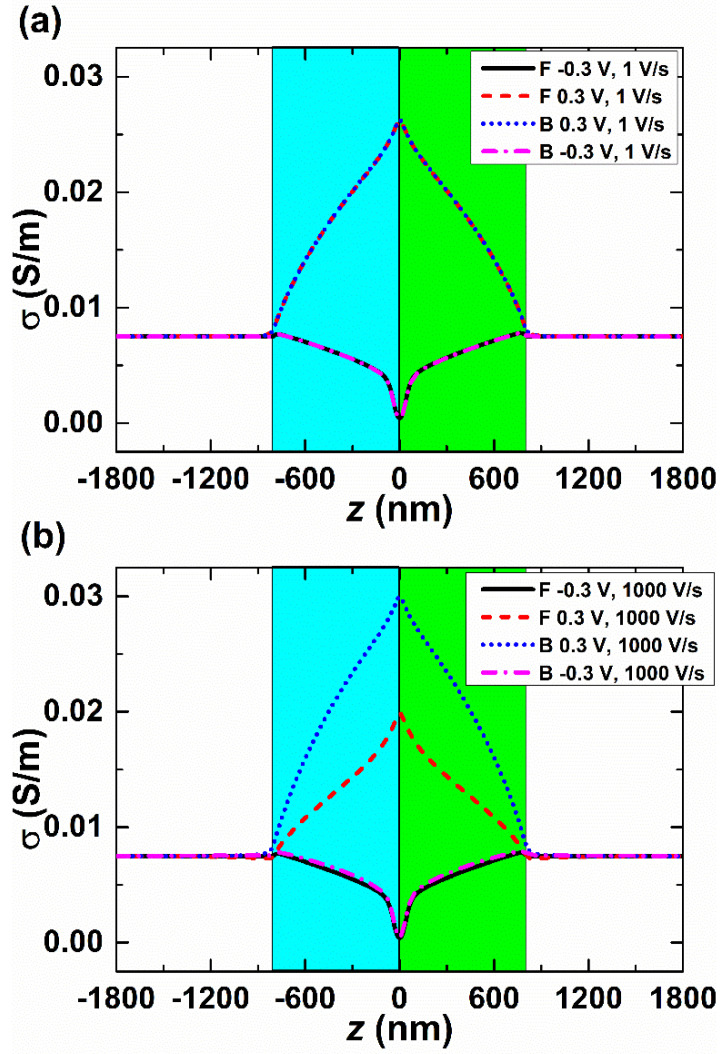
Ionic conductivity distributions, *σ*, along the *z*-axis of the bipolar interfacial nanopore at scan rates of 1 V/s (**a**) and 1000 V/s (**b**) at potential bias values Δ*ϕ* of forward −0.3 V (solid lines), forward 0.3 V (dashed lines), backward 0.3 V (dotted lines), and backward −0.3 V (dash-dotted lines). The cyan part represents the negatively charged nanochannel, and the green part represents the positively charged nanochannel. “F” and “B” represent forward and backward.

To gain further insight into the scan-rate-dependent I–V response, we plot the axial variation in the conductivity, $\sigma =\sum_{i=1}^{2}\lambda_{i}c_{i}$, in Figure 3. Here, λ*_i_* is the molar conductivity of K^+^ and Cl^−^ ions, λ_1_ = 7.352 × 10^−3^ S∙m^2^/mol and λ_2_ = 7.634 × 10^−3^ S∙m^2^/mol [46]. Figure 3 shows the ionic conductivity distributions, σ, along the *z*-axis of the bipolar interfacial nanopore at scan rates of 1 V/s (Figure 3a) and 1000 V/s (Figure 3b), with the bias potential values of ± 0.3 V. The solid and dashed lines represent the forward scan direction (from negative to positive), while the dotted and dashed-dotted lines represent the backward scan direction (from positive to negative). At a scan rate of 1 V/s, the conductivities in both forward and backward directions are identical. When the applied voltages are equal, the axial variation in conductivity aligns. However, at a scan rate of 1000 V/s, a significant difference in axial conductivity variation is observed when a positive bias potential is applied (e.g., forward 0.3 V and backward 0.3 V), whereas the difference is less pronounced for a negative bias potential (e.g., forward −0.3 V and backward −0.3 V). This observation corresponds to the I–V response results depicted in Figure 2.

Figure 3 also indicates that the conductivity at backward 0.3 V is noticeably higher than that at forward 0.3 V. This suggests that the axial variation in conductivity increases as the applied voltage transitions from forward 0.3 V to backward 0.3 V (increasing from 0.3 V to 0.5 V and then decreasing to 0.3 V). During this process, the continuous voltage change causes the number of ions in the nanopore, whether cations or anions (Figure S2), to continuously increase, resulting in a further increase in conductivity. Due to the variations in previous states, when the same 0.3 V is applied, different conductivity values are obtained. Consequently, the current is influenced not only by the bias potential that is imposed but also by the prior conditions within the bipolar interfacial nanopore, indicating that the bipolar interfacial nanopore exhibits a history-dependent behavior or has a memory effect. In the forward scan, the current follows the previous low-conductivity state, while in the backward scan, the current “memorizes” the previous high-conductivity state, leading to a higher backward current compared to the forward current.

### 3.2. Effect of the Membrane Thickness

**Figure 4:**
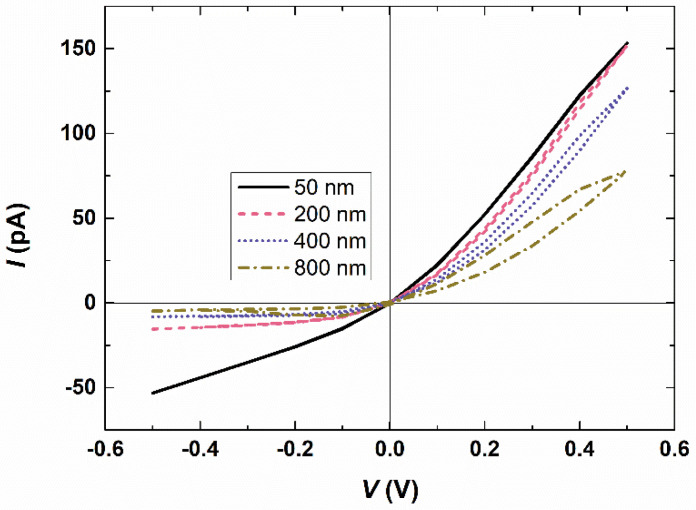
I–V response through the interfacial nanopores at a scan rate of 1000 V/s when half of the membrane thickness (height of the nanochannel) is varied from 50 nm to 800 nm. *C*_KCl_ = 0.5 mM.

Memory effects are widely recognized as resulting from the dynamic characteristics of charge carriers, such as electrons or ions, and are associated with the limited mobility of ions during their redistribution within nanopores in response to applied potentials [20]. Thus, we analyze the causes of such memory effects in bipolar interfacial nanopores. Figure 4 illustrates the impact of membrane thickness on the I–V response at a scan rate of 1000 V/s through the bipolar interfacial nanopore. The thicker the membrane, the larger the loop area, indicating that under the same voltage and different initial states, the difference in current is greater with increasing membrane thickness. This phenomenon is related to the time required for ion redistribution. The thicker the membrane, the longer it takes for ions to redistribute within the nanopore. We compare the distribution of ionic species under different conditions (Figures S2–S4). When a relatively low scan rate (e.g., 1 V/s) or a relatively thin membrane (e.g., nanochannel height of 50 nm) is used, there is no change in the distribution of ionic concentrations. In these cases, a normal current rectification behavior would be observed in the bipolar interfacial nanopore, as the ions would have enough time to reorganize inside the bipolar interfacial nanopore during the voltage scan. However, when the membrane is sufficiently thick, it produces a memory effect or a scan-rate-dependent I–V response.

### 3.3. Effect of the Salt Concentration

**Figure 5:**
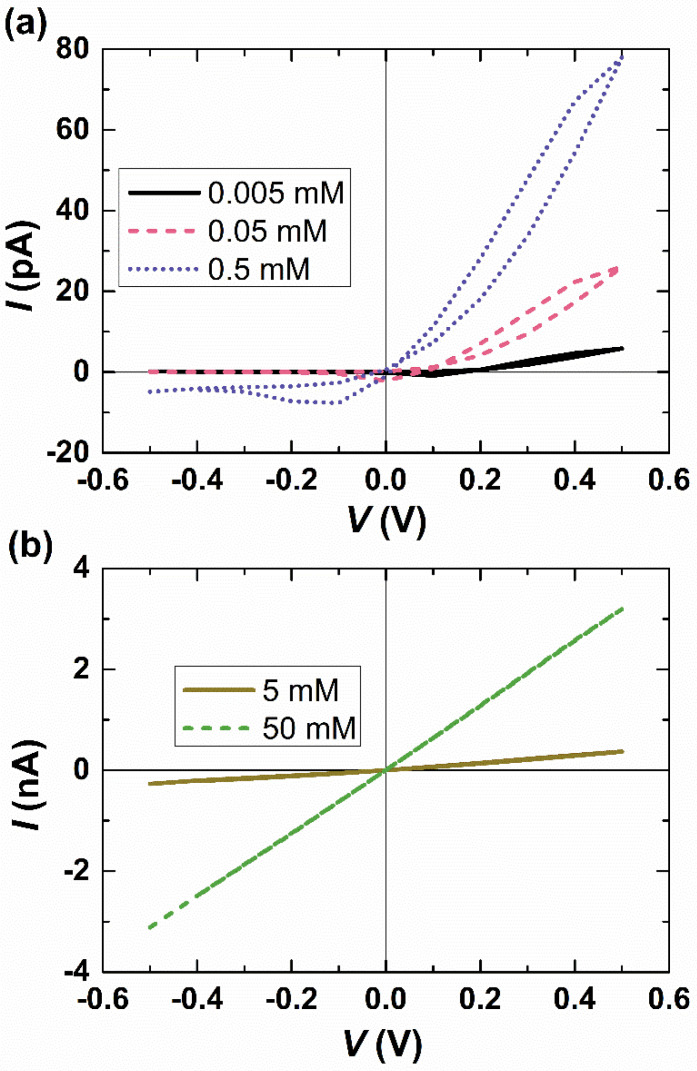
I–V responses at a high scan rate (1000 V/s) and different salt concentrations: (**a**) 0.005 mM (solid black line), 0.05 mM (dashed pink line), and 0.5 mM (dotted purple line) and (**b**) 5 mM (solid brown line) and 50 mM (dashed green line).

Figure 5 illustrates the impact of bulk salt concentration on the I–V response at a scan rate of 1000 V/s. When the bulk salt concentration is relatively low (0.005, 0.05, and 0.5 mM), we observe apparent current rectification. The cross-point potential, which separates the hysteresis loops, varies with different bulk salt concentrations and decreases as the concentration increases. This behavior is similar to that observed in single conical nanopores, attributed to the more effective screening of electrostatic interactions between ions and surface charges at higher bulk salt concentrations [20]. In 5 mM and 50 mM KCl, the I–V response of the nanopore demonstrates an almost linear relationship due to a decrease in electric double-layer thickness. According to the equation of Debye length $\lambda_{D}=\sqrt{\varepsilon_{0}\varepsilon_{f}RT/\sum_{i=1}^{2}F^{2}z_{i}^{2}c_{i}}$, the electric double-layer thicknesses are 138 nm, 43.6 nm, 13.8 nm, 4.36 nm, 1.38 nm, corresponding to KCl concentrations of 0.005, 0.05, 0.5, 5, and 50 mM, respectively. In our system, the width of the nanochannel is 40 nm. Consequently, at KCl concentrations of 5 and 50 mM, the ion current rectification is no longer present. The impact of the scan rate diminishes as the concentration increases. Similar findings were reported by Guerrette et al. [21] in experiments conducted using 10 nm diameter quartz nanopipettes in 1 M KCl, where nanopores exhibited a nearly linear I–V response at all scan rates due to a reduction in double-layer thickness. The reported concentration in their study is higher than ours as it is also influenced by other factors, such as surface charge density. The concentration at which a linear I–V response is observed increases with a decrease in the interface size of the interfacial nanopore. For instance, Figure S5 demonstrates that a 10 nm interfacial nanopore at 10 mM and 1000 V/s can still exhibit a nonlinear and scan-rate-dependent I–V response.

### 3.4. Comparison with the Conventional Nanopore

**Figure 6:**
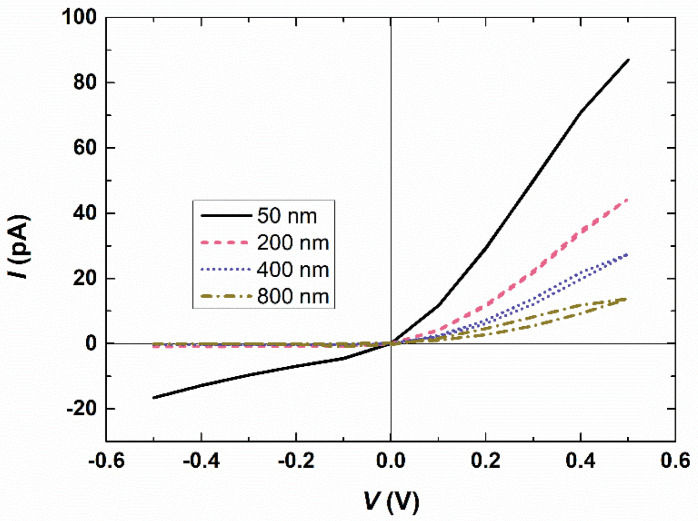
I–V response through the bipolar square nanopores when half of the membrane thickness is varied from 50 nm to 800 nm. The bipolar square nanopore and the interface of the interfacial nanopore have the same cross-sectional area. *C*_KCl_ = 0.5 mM.

As mentioned previously, the scan-rate-dependent I–V response is primarily attributed to the difference in the redistribution speed of ions compared to the voltage change. Hence, we speculate that this phenomenon is not influenced by the nanopore’s shape. To support this claim, we modeled the I–V response of a bipolar square nanopore (with a square cross-section) where the upper half carries positive charges and the lower half carries negative charges. Figure 6 presents the I–V response through the bipolar square nanopores, demonstrating that at a scan rate of 1000 V/s, the forward current curves do not coincide with the backward current curves when the length of the bipolar square nanopore is sufficient. This observation becomes more pronounced as the nanopore length increases. Thus, it is evident that conventional bipolar nanopores also exhibit memory effects when the length exceeds a certain threshold. However, we have found that the current passing through conventional bipolar nanopores, ranging from 2 × 50 nm to 2 × 800 nm in thickness, is significantly lower compared to interfacial nanopores of the same thickness. For instance, when the bias potential is 0.5 V and the cross-sectional area of the conventional bipolar nanopore matches that of the interfacial nanopore (*W*_n_ = 40 nm), the current passing through the bipolar nanopore decreases from 86.96 pA (*H*_n_ = 50 nm) to 13.76 pA (*H*_n_ = 800 nm). On the other hand, in the interfacial nanopore, the current only decreases by half, from 153.27 pA to 77.94 pA, over the same thickness range of 50 nm to 800 nm. This difference is attributed to the fact that the current in the interfacial nanopore is primarily determined by the pore size at the interface of the two nanochannels and the applied voltage, rather than the membrane thickness [43]. Therefore, the interfacial nanopore is more suitable for accurate current measurements, as low-current signals are challenging to detect.

## 4. Conclusions and Outlook

Based on a continuum-based model, we conducted a time-dependent investigation of electrokinetic ion transport in bipolar interfacial nanopores. Our study focused on examining the I–V responses of these nanopores in different concentrations of KCl solutions and at various scan rates. The findings reveal that the I–V response in bipolar interfacial nanopores is not solely dependent on the applied voltage but also influenced by the previous state at high scan rates, indicating the presence of memory effects. We observed interesting hysteresis loops and a nonzero cross-point potential in bipolar interfacial nanopores when the bulk salt concentrations were relatively low, within specific scan rates and potential ranges. However, at higher bulk salt concentrations, the scan-rate-dependent I–V response vanished, and the nanopores exhibited nearly ohmic behavior. Furthermore, we demonstrated that the scan-rate-dependent I–V response can also be observed in conventional bipolar nanopores. This behavior arises because, at high scan rates, the mass transfer of ionic species lags behind the timescale of the voltage scan. However, compared to conventional bipolar nanopores, bipolar interfacial nanopores can induce a larger ionic current, making them easier to detect. These findings lay the groundwork for the potential application of bipolar interfacial nanopores in memristors.

## Data Availability

The data that support the findings of this study are available from the corresponding authors upon reasonable request.

## Supplementary Materials

The following supporting information can be downloaded at https://www.mdpi.com/article/10.3390/mi15091176/s1, Table S1: Boundary conditions; Figure S1: The nanochannel area carrying surface charge; Figure S2: Distribution of cations and anions along the axis of the bipolar interfacial nanopore when *H*_n_ = 800 nm, *C*_KCl_ = 0.5 mM, and *v* = 1000 V/s; Figure S3: Distribution of cations and anions along the axis of the bipolar interfacial nanopore when *H*_n_ = 800 nm, *C*_KCl_ = 0.5 mM, and *v* = 1 V/s; Figure S4: Distribution of cations and anions along the axis of the bipolar interfacial nanopore when *H*_n_ = 50 nm, *C*_KCl_ = 0.5 mM, and *v* = 1000 V/s; Figure S5: I–V response when *W*_n_ = 10 nm, *C*_KCl_ = 10 mM, and *v* = 1000 V/s.
