# Supplementary material for: Scan-Rate-Dependent Ion Current Rectification in Bipolar Interfacial Nanopores
Source: Micromachines (Basel). 2024 Sep 23;15(9):1176. doi: 10.3390/mi15091176 (PMC11433788; doi:10.3390/mi15091176)
Supplement: Supplementary file 1 [file micromachines-15-01176-s001.zip › micromachines-3210572-supplementary.pdf]

micromachines

MDPI

Supplementary Material

Scan-Rate-Dependent Ion Current Rectification in Bipolar Interfacial Nanopores

Xiaoling Zhang [1,*], Yunjiao Wang [2], Jiahui Zheng [3], Chen Yang [3], and Deqiang Wang [2,*]

[1] School of Smart Health, Chongqing Polytechnic University of Electronic Technology, Chongqing 401331, China
[2] Chongqing Institute of Green and Intelligent Technology, Chinese Academy of Sciences, Chongqing, 400714, China; wangyunjiao@cigit.ac.cn (Y. W.)
[3] Key Laboratory of Biorheological Science and Technology, Ministry of Education and Bioengineering College, Chongqing University, Chongqing 400044, China; jiahuizheng@cqu.edu.cn (J.Z.); yangchencq@cqu.edu.cn (C.Y.)
* Correspondence: zhangxiaoling@cqcet.edu.cn (X. Z.); dqwang@cigit.ac.cn (D. W.)

Table S1. Boundary conditions.

Surface	Poisson	Nernst–Planck	Navier–Stokes
End of the lower reservoir	$\phi = \Delta\phi$	Concentration ($c_i = C_{i0}$)	Pressure = 0
End of the upper reservoir	Ground	Concentration ($c_i = C_{i0}$)	Pressure = 0
The walls of the nanopore	Surface charge density of upper nanochannel<br>$-\mathbf{n}\cdot\nabla\phi = \sigma_{w1}/(\varepsilon_0\varepsilon_f)$<br>Surface charge density of lower nanochannel<br>$-\mathbf{n}\cdot\nabla\phi = \sigma_{w2}/(\varepsilon_0\varepsilon_f)$	No flux<br>$\mathbf{n}\cdot\boldsymbol{N}_i = 0$	No slip
The walls of the reservoirs	Zero charge<br>$\mathbf{n}\cdot\nabla\phi = 0$	No flux<br>$\mathbf{n}\cdot\boldsymbol{N}_i = 0$	Slip
Planes of symmetry		Symmetry	Symmetry

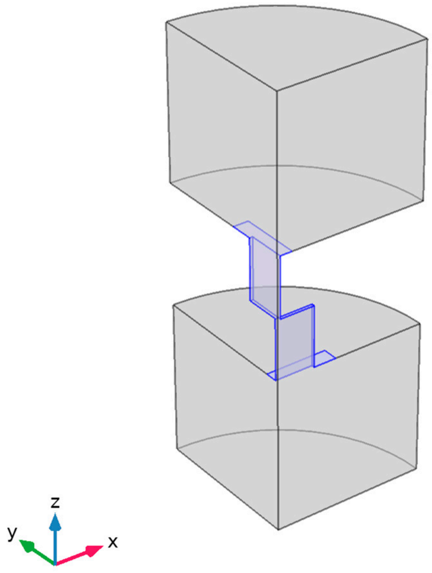

Figure S1. The nanochannel area carrying surface charge.

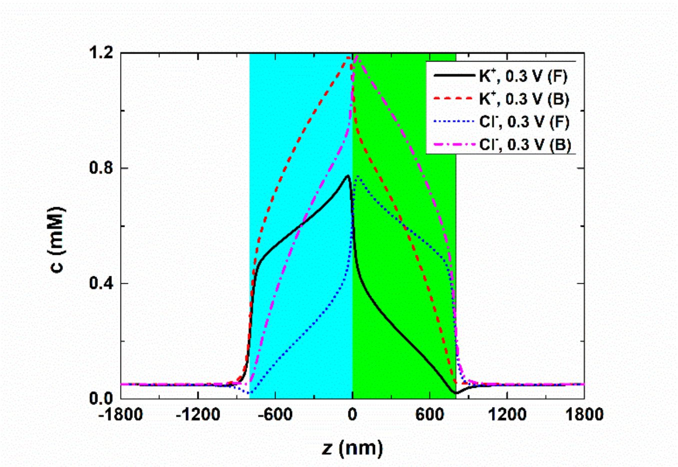

Figure S2. Distribution of cations and anions along the axis of the bipolar interfacial nanopore when H_n = 800 nm, C_{KCl} = 0.5 mM, and v = 1000 V/s.

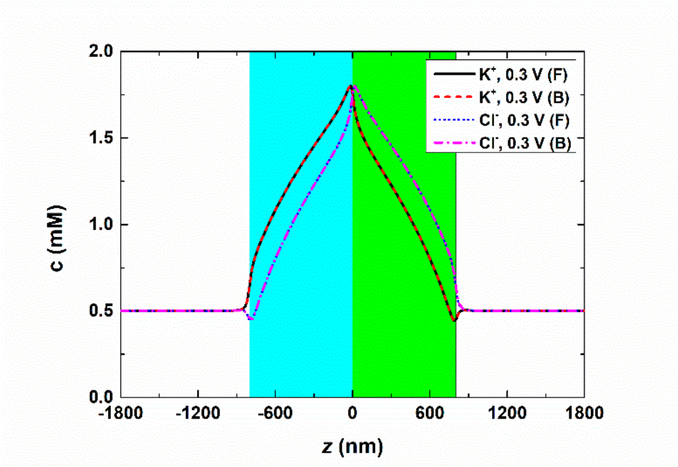

Figure S3. Distribution of cations and anions along the axis of the bipolar interfacial nanopore when H_n = 800 nm, C_{KCl} = 0.5 mM, and v = 1 V/s.

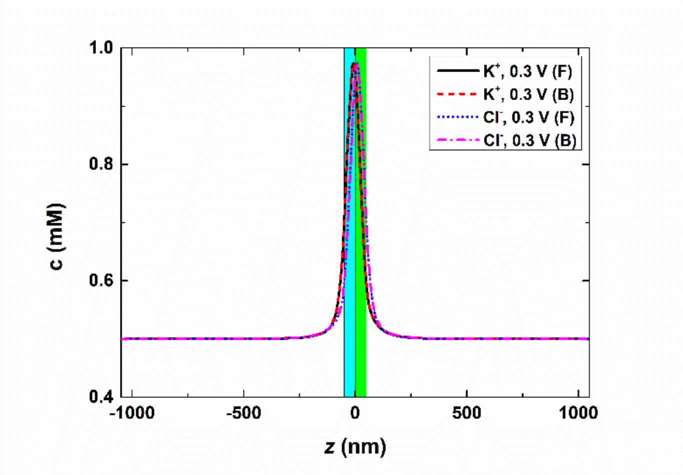

Figure S4. Distribution of cations and anions along the axis of the bipolar interfacial nanopore when $H_n = 50$ nm, $C_{KCl} = 0.5$ mM, and $v = 1000$ V/s.

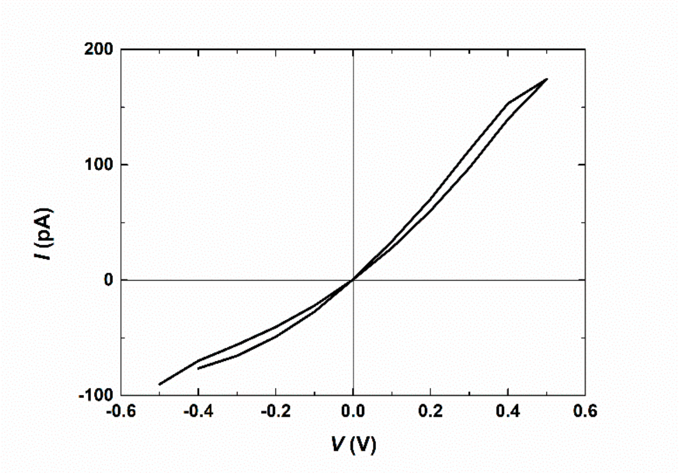

Figure S5. I–V response when $W_n = 10$ nm, $C_{KCl} = 10$ mM, and $v = 1000$ V/s.
